# Population, Ecological Footprint and the Sustainable Development Goals

**DOI:** 10.1007/s10640-021-00595-5

**Published:** 2021-11-16

**Authors:** Partha Dasgupta, Aisha Dasgupta, Scott Barrett

**Affiliations:** 1 Faculty of Economics, University of Cambridge, Cambridge, UK; 2 Foreign, Commonwealth, and Development Office, Abuja, Nigeria; 3 School of International and Public Affairs, Columbia University, New York, USA

**Keywords:** Biosphere, Ecological footprint, Sustainable development goals, Impact inequality, Natural regeneration rate, Population

## Abstract

The Anthropocene can be read as being the era when the demand humanity makes on the biosphere’s goods and services—humanity’s ‘ecological footprint’—vastly exceeds its ability to supply it on a sustainable basis. Because the ‘ecological’ gap is met by a diminution of the biosphere, the inequality is increasing. We deploy estimates of the ecological gap, global GDP and its growth rates in recent years, and the rate at which natural capital has declined, to study three questions: (1) at what rate must efficiency at which Nature’s services are converted into GDP rise if the UN’s Sustainable Development Goals for year 2030 are to be sustainable; (2) what would a sustainable figure for world population be if global living standard is to be maintained at an acceptably high level? (3) What living standard could we aspire to if world population was to attain the UN’s near lower-end projection for 2100 of 9 billion? While we take a global perspective, the reasoning we deploy may also be applied on a smaller scale. The base year we adopt for our computations is the pre-pandemic 2019.

## The Global Economy in the Anthropocene

**Fig. 1:**
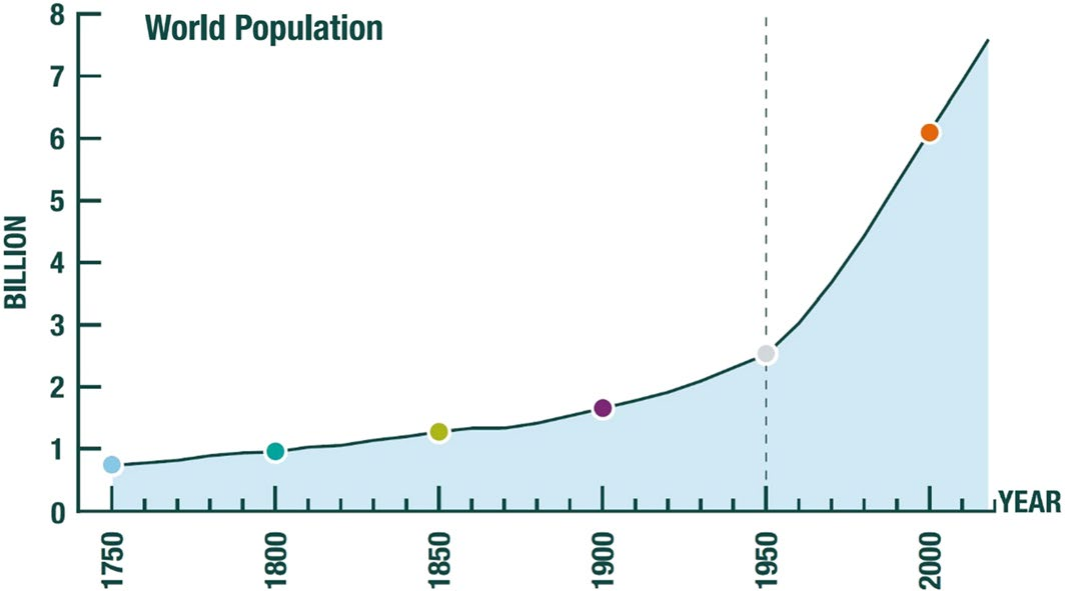
Global Population since 1750 CE. *Source*: UNPD (2019)

**Fig. 2:**
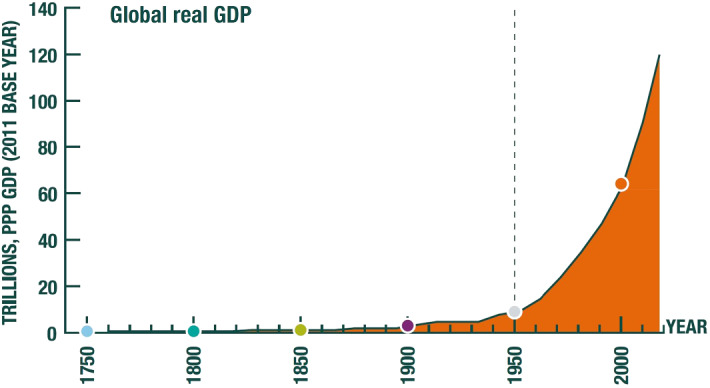
Global Real GDP since 1750 CE. *Source*: Our World in Data based on World Bank and Maddison Project Database, version 2018. Bolt et al. (2018), “Rebasing ‘Maddison’: new income comparisons and the shape of long-run economic development”, Maddison Project Working paper 10

**Fig. 3:**
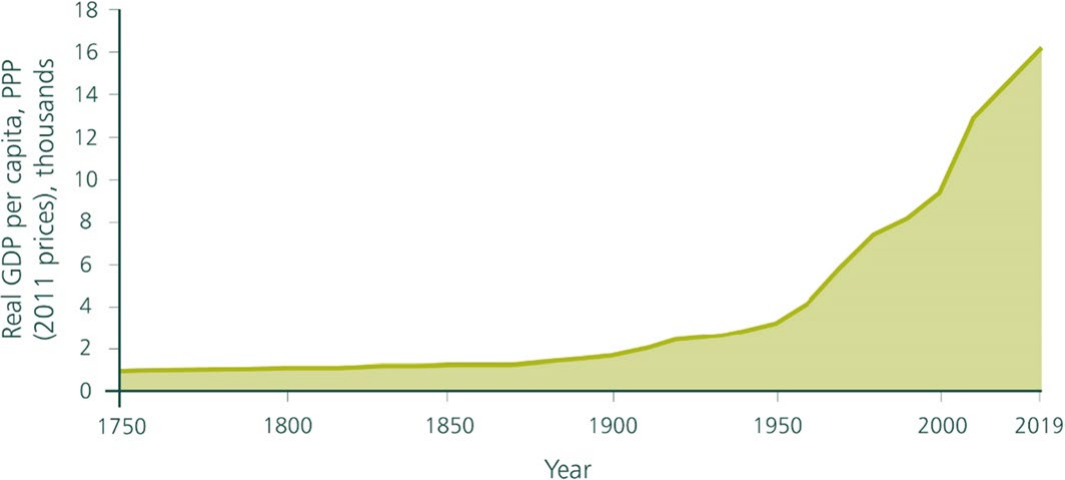
Global real GDP per Capita since 1750 CE

World population in 1950 was approximately 2.5 billion (Fig. 1) and world output of final goods and services (i.e., global GDP) at 2011 prices a bit over 8.2 trillion dollars at purchasing price parity, PPP—henceforth, ‘dollars’—(Fig. 2). The average person’s annual income was thus about 3300 dollars (Fig. 3), a high figure by historical standards. Since that time, the world has prospered beyond recognition. Life expectancy at birth in 1950 was 46, today it is above 72. The proportion of the world’s population living in extreme poverty (currently taken to be 1.90 dollars) has fallen from nearly 60% in 1950 to less than 10% today. In 2019 the global population had grown to over 7.7 billion (Fig. 1) even while global GDP per capita had risen to nearly 16,000 dollars (at 2011 prices; Fig. 3). World GDP was a bit above 120 trillion dollars (at 2011 prices), meaning that globally measured economic activity had increased more than 14-fold in only 70 years, something that had not remotely been experienced before (Fig. 2).

This extraordinary achievement has, however, come at a life-threatening price, one which ecologists and Earth scientists have been pointing to for some time. We are causing species extinction at 100–1000 times the average extinction rate over the past several millions of years (the ‘background rate’) of 0.1–1 per million species per year, and the rate is continuing to rise. Allied to species extinction, the biosphere is being so degraded by human activities, that several of vital regulating and maintenance services (Sect. 2) we enjoy from it are increasingly threatened, the most prominent in the public eye being the stable climate in which our economies have evolved. A proposal by Earth scientists (Waters et al. 2016; Voosen 2016) that mid-twentieth century should be regarded as the time we entered a new, human dominated era—the Anthropocene—matches Figs. 1, 2, 3 exactly.^1^

## Ecological Footprint and the Impact Inequality

In this paper we view the biosphere as a mesh of interconnected ecosystems that differ in spatial scales and operate at different speeds. The biosphere is a self-organising regenerative entity.^2^ The Anthropocene can thus be read as being the era when the demand humanity makes of the goods and services produced by the biosphere in a period exceeds its ability to meet it on a sustainable basis. We call the (ecological) gap between demand and sustainable supply the *Impact Inequality*.^3^ One reason the gap has not been noted in the received macroeconomics of growth and development and the economics of poverty is that both subjects have neglected to include ecosystems in an essential way in their accounts. Gross domestic product (GDP) and its distribution reflect gross incomes, they do not account for the diminution of ecosystems when overstretched by human activities. GDP is a flow, in contrast to ecosystems, which are stocks. National governments should now include in the statistics they compile a record of inventories of stocks—*produced* capital (roads, building, machines, ports), *human* capital (population, health, and education), and *natural* capital (wetlands, woodlands, mangroves, forests, plantations and agricultural land, peatlands, and fossil fuels and minerals). In what follows we build our account of sustainable development in the language familiar to *asset managers* (Dasgupta 2021) and study the composition of assets and the flow of goods and services they provide in the contemporary world.

### Global Demand

In a classic paper Ehrlich and Holdren (1971) called the demand humanity makes on Nature’s goods and services in a period (e.g., a year) our *Impact* on the biosphere. Today it is common to call Impact our global *ecological footprint*.^4^ Humanity’s footprint includes not only what we harvest and draw upon from Nature, but also the services Nature offers for accommodating our waste (e.g., recycling nutrients). We may then view Nature’s services through a common lens and regard pollution to be the reverse of conservation. The idea is to track the effects of human activities on the biosphere from source to sink.

As Impact is caused by our activities, we first need a measure of our activities and then find a conversion factor to map them into ecological footprint. Global GDP is probably the closest we can get to a quantitative measure human activities per period, so we use that as our measure. It then proves useful to decompose global GDP, as Ehrlich and Holdren did, into global population size and global GDP per capita (Ehrlich and Holdren called the latter, ‘affluence’). Let *N* denote the former and *y* the latter. Global GDP is then *Ny*, expressed in dollars. Now let α be a numerical measure of the efficiency with which Nature’s goods and services are converted by humanity into global GDP. Armed with these three factors that together make up the global ecological footprint, we may express it as *Ny*/α.

Each of the factors affects the other two, and they in turn depend on both technology and institutions. Thus, the invention of the chain saw, bull dozers, and modern fishing trawlers and the gears that come with them have made resource extraction cheaper, meaning that they have led to an increase in *y* (and a corresponding diminution of the biosphere, Sect. 2.2); advances in clean energy technology and institutional reforms that lead to reductions in food waste would raise α; and investment in women’s education and modern family planning and reproductive health can be expected to lower future *N* and thereby increase future *y*.^5^

### Global Supply

Set against the global ecological footprint is the biosphere’s supply of goods and services. The Common International Classification of Ecosystem Services (CICES), which also identifies the contributions ecosystems make to human well-being, is built on the pioneering work of the Millennium Ecosystem Assessment (MEA 2005a, b, c, d). It consists of three categories of ecosystem services, contributing directly or indirectly to human well-being. They are:

#### Provisioning Services (PS)

This category includes the provision of materials and energy needs for the range of products we obtain from ecosystems. It includes food, fresh water, fuel (dung, wood, and leaves), fibre (grasses, timber, cotton, wool, silk), biochemicals and pharmaceuticals (medicines, food additives), genetic resources (genes and genetic information used for plant breeding and biotechnology), and ornamental resources (skins, shells, flowers).

#### Regulating and Maintenance Services

This category regulates and maintains ecosystem processes, including maintaining the gaseous composition of the atmosphere, regulating both local and global climate (temperature, precipitation, winds and currents), controlling erosion (retaining soil and preventing landslides), regulating the flow of water (the timing and magnitude of runoff, flooding, and aquifer recharge), purifying water and decomposing waste, regulating diseases (controlling the abundance of pathogens such as cholera and disease vectors such as mosquitoes), controlling crop/livestock pests and diseases, pollinating plants, and offering protection against storms (forests and woodlands on land, mangroves and coral reefs on coasts), recycling nutrients, and maintaining primary production and oxygen production through photosynthesis.

#### Cultural Services

This category offers non-material benefits, including spiritual experiences and an identification with religious values. It is perhaps more appropriate to trace these experiences and values to Nature, rather than ecosystems, because the latter is a term of recent origin. People find aesthetic value in Nature, which gives expression in private gardens and public parks and protected areas (forests and coast lines). Ecosystems influence social relationships (social capital in coastal fishing villages take a different form from social capital in nomadic herding and agricultural societies). The local ecosystem offers people a sense of place, their cultural landscape. And there are ecosystems that attract tourism and recreation.

#### Ecological Dynamics

Provisioning, regulating/maintenance, and cultural services amount to a multiplicity of goods and services per period. It pays to let our imagination soar and suppose it is possible to estimate their accounting prices and arrive at an aggregate scalar measure, *G*, expressed in dollars. *G* is a numerical measure of Nature’s regenerative rate. Let *S* denote an aggregate scalar measure of the biosphere, viewed as a stock of natural capital. *S* is also assumed to be measured in accounting prices. We write *G* = *G*(*S*).

The biosphere is bounded. For simplicity, we extend the basic formulations of fisheries, soil, and forests, and suppose *G* is an increasing function of *S* (i.e., d*G*/d*S* > 0) for small *S* and a decreasing function of *S* (i.e., d*G*/d*S* < 0) for large *S*.^6^ Investment in Nature conservation and restoration increases *S*, while advances in biotechnology (e.g., genetically modified crops) raise the *G*-function. In contrast, innovations that reduce the cost of harvesting Nature result in a diminution of *S*.

Humanity’s ecological footprint does not have to equal *G*, because the difference would be met by a change in the biosphere’s stock, *S*. Thus, if *R* is the rate at which humanity harvests and draws on Nature (i.e., our ecological footprint) and *t* is date (assumed continuous), then

1 $$ {\text{d}}S\left( t \right)/{\text{d}}t = G\left( {S\left( t \right)} \right) \, {-}R\left( t \right) $$

**Fig. 4:**
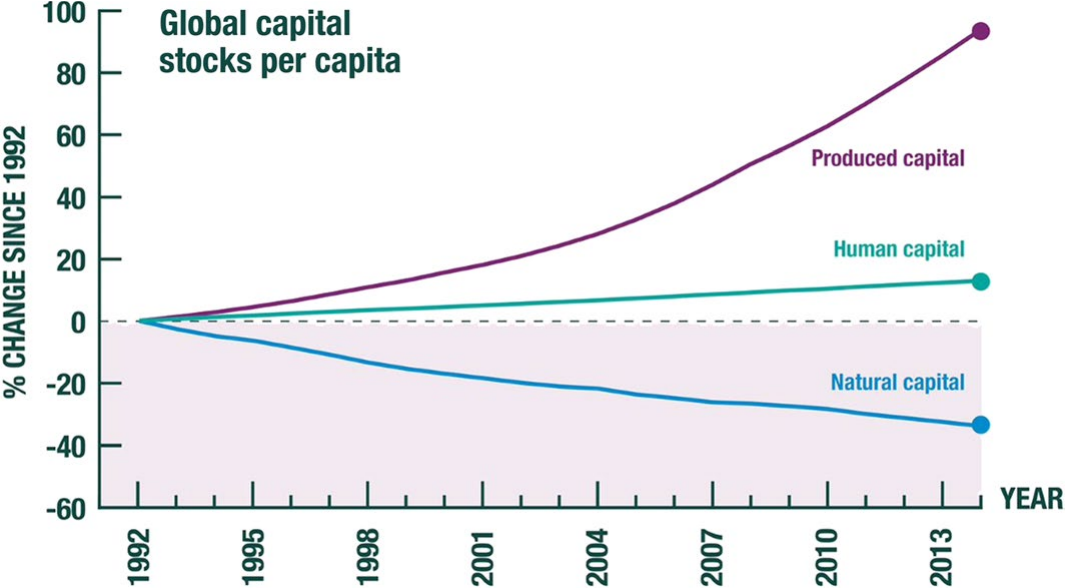
Global Wealth per Capita, 1992–2014. *Source*: Managi and Kumar (2018)

A world rich in healthy ecosystems could on Utilitarian grounds choose to draw down the biosphere (i.e., natural capital) somewhat and use the goods and services it supplies for accumulating produced capital and human capital. That is what economic development has come to mean among many people. Although that may not have mattered in decades past, it matters today, because the process of economic development humanity has chosen to follow has led to an alarming diminution of natural capital. For example, in a publication sponsored by the UN Environment Programme (Managi and Kumar 2018), the authors have tracked produced capital, human capital and natural capital over the period 1992–2014 in 140 countries. Their estimates are that produced capital per head doubled and human capital per head increased by about 13%, but the value of the stock of natural capital per head (forests, fisheries, minerals) declined by nearly 40% (Fig. 4). Note though that the trend displayed in Fig. 4 cannot be maintained, because it involves depleting *S*. If the trend were to continue, *G* would collapse in due course, even as the biosphere tips over into a state wholly unexperienced in the human economy.^7^

### Trade-offs Between Ecological Services

It is significant that the processes governing regulating and maintenance services are either several steps removed from our direct experience or are felt by us only over the long run. The services themselves are mostly hidden from view and are also mostly silent (think of the bewildering number of activities in operation in the soils and in the waters). In contrast, provisioning and cultural services have readily detectable outcomes or are directly observable and can be felt even in the short run (e.g., in agricultural production, water deployment, forestry, fishing).

The competition humanity has created between the availability of provisioning services on the one hand and regulating and maintenance services on the other is vividly illustrated by tropical rainforests. These ecosystems produce goods such as fuelwood, fodder, timber, leaf manure, food, medicines, and supply such services as sequestering carbon, offering a habitat for wildlife, and more generally housing biodiversity. Upstream forests also regulate waterflow (e.g., groundwater recharge, flood control) and conserve soil. The competing demands are a reason it is easy to overlook the significance of regulating and maintenance services; they are easy prey to economic development in the form the latter has been pursued over the years. Economic development has come to mean growth in the products we enjoy from provisioning services and such cultural services as tourism. But the pursuit of economic growth (i.e., growth in GDP) has led to a decline in the ability of the biosphere to supply regulating and maintenance services. That decline endangers the biosphere’s ability to supply provisioning, maintenance, and cultural services to our descendants.

### The Impact Inequality

*Ny*/α is our proxy measure of the global ecological footprint (denoted as *R* in Eq. (1)). If the footprint exceeds the biosphere’s regenerative rate *G*, the stock *S* diminishes. Similarly, if the footprint is less than the biosphere’s regenerative rate, the stock increases.^8^ To illustrate, establishing and enforcing well-designed property rights to natural capital that was previously an open-access resource raise α and result in a reduction in extracted output, so that *y*/α declines (Dasgupta 2021: Ch. 6). That reduces the gap between demand and supply, which in turn raises future *S* from what it would otherwise be (Eq. (1)); which in its turn further reduces the gap. In contrast, the gap between *Ny*/α and *G* has been increasing in the Anthropocene, even as *S* has been diminishing.

**Fig. 5:**
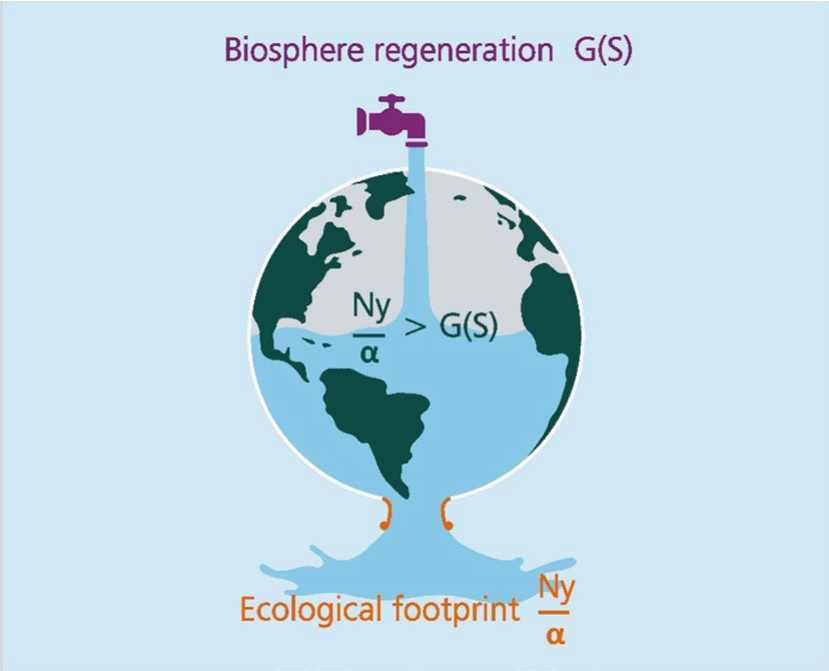
The impact inequality. *Source*: Dasgupta (2021)

We call the gap between demand and sustainable supply the *Impact Inequality* (Barrett et al. 2020; Dasgupta 2021; Fig. 5), and it reads as

2 $$ Ny/\alpha \, > G\left( S \right) $$

In what follows we deploy the Impact Inequality to address three questions: (1) At what rate must α increase if the UN’s Sustainable Development Goals (SDGs)—see below—for year 2030 are to be sustainable? (2) What would a sustainable figure for global population *N* be if global GDP per head *y* is to be maintained at an acceptable level? (3) What living standard could we aspire to if global population was to attain the UN’s near lower-end projection for 2100 of 9 billion?

The questions are studied sequentially in the following two sections. Our estimates are constructed out of very crude data, which is why the calculations we offer are crude, back-of-the-envelope exercises. We cannot do better because the applied economics of sustainable development remains an underdeveloped field of enquiry. Moreover, growth and development economics and the economics of poverty continue to keep Nature in the side-line. So, there is little to build on. We are obliged to work with point estimates because the data are so sparse in this field of macroeconomic enquiry that we have no way of placing error bars round them. Our purpose is to point to ways in which the three questions can be addressed, nothing more. That the estimates we offer in this paper are crude is not an admission, it is an assertion.

## The Sustainability of the UN’s Sustainable Development Goals

**Fig. 6:**
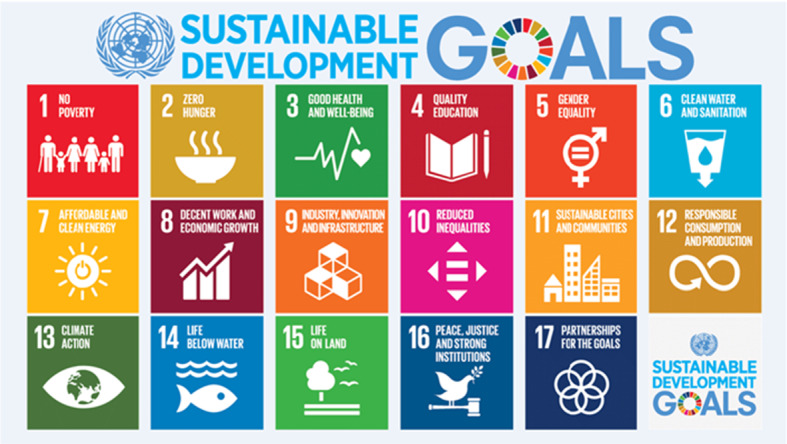
The UN’s Sustainable Development Goals. *Source*: United Nations (2015)

In September 2015, the United Nations General Assembly agreed on an agenda for sustainable development in member countries. Nations committed themselves to meeting 17 Sustainable Development Goals by year 2030 (Fig. 6). Structurally the Goals are not independent of one another (Barbier and Burgess 2019), meaning that they need to be addressed simultaneously. The SDGs involve 169 socio-economic targets. To measure progress in meeting those targets, it was proposed to track more than 240 socio-economic indicators over the coming years.

International agreement on the SDGs was a remarkable, even noble, achievement, for the Goals unpick features of lives that, if improved, would enable us to live well. But as there is mutual dependence among the Goals, it is a puzzle that their announcement was not accompanied by an examination of whether, assuming they are achieved, the Goals are sustainable. Because we are now in a situation where there is an ever-growing Impact Inequality, sustainability should as a bare minimum require that the inequality be converted into an equality. To be sure, COVID-19 will have had a serious effect on the possibility of reaching the Goals by 2030.^9^ But as the pandemic is not yet over, we study the sustainability of the SDGs from before its occurrence. The vantage point we adopt is 2019.

### Raising α to Close the Impact Inequality

So then, how large is the current overshoot of *Ny*/α over *G*? The Global Footprint Network (GFN) defines ecological footprint not as *Ny*/α, but as the ratio of *Ny*/α to *G*(*S*). Summarising GFN’s work, Wackernagel and Beyers (2019) estimated that the ratio increased from 1 in the late 1960s to approximately 1.7 in 2019. The authors interpreted the number 1.7 as saying that we need 1.7 Earths to supply our current ecological demands on a sustainable basis.^10^ A rise from 1 to 1.7 over a 50-year period means that the ratio increased at an average annual rate of 1.1%. Meanwhile, global GDP (*Ny*) at constant prices has increased since 1970 at an average annual rate of 3.4%.

We turn to the right-hand side of the Impact Inequality. Managi and Kumar (2018) estimated that the value of per capita global natural capital declined by 40% between 1992 and 2014 (Fig. 4). That converts to an annual percentage rate of decline of 2.3%. But world population grew at approximately 1.1% in that period. Taken together it follows that the value of global natural capital declined at an annual rate of 1.2%. Because there are no estimates of the form of the *G*-function, we follow GFN in assuming that local variation is a good approximation, meaning that *G* is proportional to *S*. So, *G* can also be taken to have declined at an annual rate of 1.2%.^11^

The estimates for the annual percentage rates of change of *Ny*, *G*, and [*Ny*/α]/*G* enable us to calculate that α had been increasing at an annual percentage rate of 3.5% in the period 1992 to 2014. Suppose we want to reach Impact Equality in year 2030. That would require [*Ny*/α]/*G* to shrink from its value of 1.7–1 in 10 years’ time, implying that it must decline at an average annual rate of 5.4%. Assuming global GDP continues to grow at 3.4% annually and *G* continues to decline at 1.2% (i.e., business is assumed to continue as usual), how fast must α rise?

To calculate that, let us write as g(*X*) the percentage rate of change of any variable *X*. We then have

3 $$ {\text{g}}([Ny/\alpha ]/G) = {\text{g}}\left( {Ny} \right){-}{\text{g}}(\alpha ){-}{\text{g}}\left( G \right) $$

Equation (3) can be re-arranged as

4 $$ {\text{g}}(\alpha ) = {\text{g}}\left( {Ny} \right){-}{\text{g}}([Ny/\alpha ]/G){-}{\text{g}}\left( G \right) $$

We now place the estimates of the terms on the right-hand side of Eq. (4) to obtain

$$ {\text{g}}(\alpha ) = 0.0{34} + 0.0{54} + 0.0{12} = 0.{1} $$

In short, α must increase at an annual rate of 10%. As that is a huge hike from the historic rate of 3.5%, we consider a different scenario.

Suppose global GDP was to remain constant in real terms from now to year 2030 and draconian steps were taken by us over our demands to limit the rate of deterioration of the biosphere to an annual 0.1%. What would be the required rate of increase in α need to be? Using Eq. (4) we have g(α) = 0.054 + 0.001 = 5.5%. Even that is considerably larger than the 3.5% rate at which α has been increasing in recent decades. We should conclude that it is more than doubtful that SDGs can be attained on a sustainable basis in 2030 if we were to rely only on efficiency gains.

### Income Inequality and the Global Ecological Footprint

We have been framing the problem of sustainable development in entirely aggregate terms. How does the distribution of incomes affect the Impact Inequality? More particularly, how does the global income distribution affect the global ecological footprint?

**Fig. 7:**
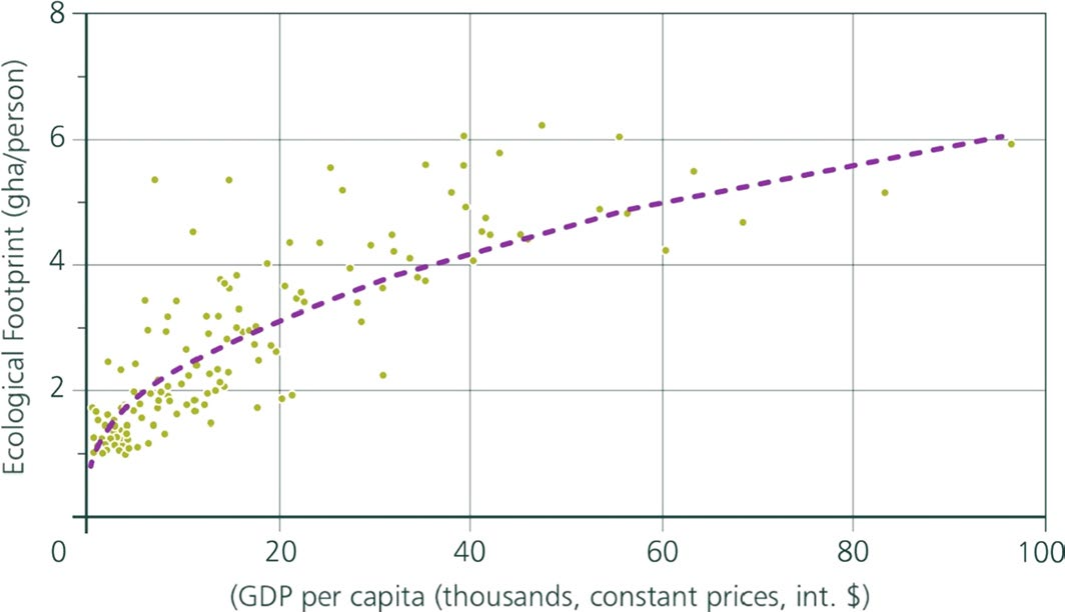
Ecological footprint and income. *Note* Data taken from World Bank (2018) on per capita GDP of nations (expressed in thousands of international dollars, at 2017 prices) and estimates from the Global Footprint Network (Wackernagel and Beyers 2019) on ecological footprint per capita (in global hectares). A total of 136 countries are in the sample, including both developed and developing countries. If *I* denotes ecological footprint and y is income, the estimated functional form is *I* = 0.9717y^0.4141^, with R^2^ = 0.7067. *Source*: Dasgupta (2021, Fig. 13.1.7)

IPCC (2014) reported from cross national statistics that carbon emissions are an increasing function of income. There is a corresponding finding that says ecological footprint is an increasing function of income (Wackernagel and Beyers 2019). A commonplace intuition would have it that because higher incomes are associated with a larger ecological footprint, egalitarian redistribution of incomes (i.e., from the rich to the poor) would reduce the Impact Inequality.^12^ Dasgupta and Dasgupta (2017) observed instead that if ecological footprint is a (strictly) convex function of income, then egalitarian redistributions of incomes—holding aggregate income fixed—would indeed reduce the global ecological footprint; but if it is a (strictly) *concave* function, the reverse holds. In the Appendix we use a recent estimate of the functional relationship between income and ecological footprint (taken from Dasgupta 2021) to find that the relationship is concave (Fig. 7).

The finding is depressing, for it says there is a tension between intergenerational and intragenerational equity. It says that if we wish to commend more egalitarian distributions of global income today without further increasing the Impact Inequality, there needs to be a reduction in global GDP from what it is. Put another way, a global GDP with inequality in incomes is larger than the corresponding ‘Impact-equivalent’ global GDP.

## Complementarities Between Produced and Natural Capital: The Population-Consumption Trade-off

Because of the urgency created by the overshoot of our demands on the biosphere, we lay stress on the fact that natural capital is a *complementary* factor to produced capital and human capital. Their complementarity is an inevitable feature of our production system because material must balance. What is produced returns to the Earth system, where it is then assimilated: the waste is either decomposed or, as in the case of persistent pollutants, accommodated. Either way, it makes a further demand on the biosphere. Even though an additional unit of either produced capital or human capital would raise final output, unless it accompanies resource saving technological progress (rise in α) the additional waste would inevitably result in an increase in the demand made of the biosphere.^13^ We use that to guide our analysis.

In what follows we deploy the idea of Impact-equivalent GDP and return to aggregate reasoning. Our decomposition of humanity’s ecological footprint, *Ny*/α, could appear to show that substitution possibilities between *N* and *y* are reflected as a product of *N* and *y*, that is, they take the form of rectangular hyperbolae. Arithmetically that is true, but *y* is not independent of *N*, it is a function of *N*; for we humans are not merely consumers, we are born with hands and a brain. In short, *y* = *y*(*N*). In standard models of economic growth, d*y*/d*N* < 0, but the functional form is not that of a rectangular hyperbola.^14^ We confirm that below in a simple, reduced-form model and ask the remaining two questions we listed previously: (2) How many people can Earth support sustainably at an acceptable living standard, given today’s technology? (3) What living standard could we aspire to if global population was to attain the UN’s near lower-end projection for 2100 of 9 billion? In the exercises that follow, we assume that the efficiency factor α remains the same as it is today. To extend the calculations with alternative values of α would be a routine exercise, and we show how that can be done in a simple manner.

### How Many People Can Earth Support in Comfort?

Per capita global GDP in 2019 was approximately 16,000 dollars at 2011 prices. For answering (2), we use the figure of 20,000 (international) dollars at 2011 prices. As the figure falls in the range of per capita incomes in the World Bank’s list of high middle-income countries, we use it to represent an acceptable standard of living.^15^

We assume that people apply their labour on produced capital and the biosphere's goods and services to produce an all-purpose commodity that can be consumed. As of now we have little quantitative knowledge of the biosphere's dynamics when viewed in the aggregate, that is, we have no estimates of the *G*-function. But as produced capital and natural capital are complements of one another, an expansion of the stock of produced capital depresses the stock of natural capital.^16^ Rockström et al. (2009) have found evidence in the Earth system’s signatures that the planet is today so stretched in its ability to supply regulating and maintenance services, that further deteriorations would take it into unchartered terrains in its boundaries. So, we now regard *K* to be an aggregate measure of natural capital and produced capital and hold it fixed to ensure that there is no further deterioration of the biosphere. The idea is to stop *K* on its tracks by a global quota on what we are permitted to take from the biosphere. To illustrate, quotas are applied routinely to fisheries and forestry, and for access to potable water in dry regions. The recent international agreement to limit the rise in mean global temperature to 1.5 °C above what it was in pre-industrial times is tantamount to the use of quotas in emissions. Wilson (2016) has made an impassioned plea to leave half of Earth free of human encroachment. We follow that route to identify a sustainable socio-ecological state.

Let *Q* be aggregate output. If global population is *N* and φ the proportion of *N* in production, we assume that

5 $$ Q = K^{{({1} - \rho )}} [\varphi N]^{\rho } ,\quad 0 \, \le \, \rho \, < { 1}, \, 0 \, < \, \varphi \, < { 1} $$

We now estimate *K*^(1−ρ)^ (Eq. 5) from the current size of the world economy.

We assume that the value of the world's production of final good and services draws proportionately on ecosystem services at all levels.^17^ In 2019 world output was about 120 trillion dollars at 2011 prices. Using the model of production in Eq. (5), we therefore have

6 $$ K^{{{1} - \rho }} \left[ {\varphi N} \right]^{\rho } = {12}0{\text{ trillion dollars}} $$

World population was 7.8 billion in late 2019. The global dependency ratio, that is, the ratio of the sum of the number of people below age 15 and above age 65 to the number of people between 15 and 65, is today about 1.6–1. Thus φ = 1/2.6, and so φ*N* = 3 billion. A huge empirical literature in economics suggests that as a rounded figure, ρ = 0.5 is not unreasonable. Equation (6) then says

7 $$  \begin{aligned}   K^{{0.{\text{5}}}}  =  & {\text{12}}0 \times {\text{1}}0^{{{\text{12}}}} /\left( {{\text{3}} \times {\text{1}}0^{{\text{9}}} } \right)^{{0.{\text{5}}}} {\text{dollars per producer}}^{{0.{\text{5}}}}  \\    ~ \approx  & {\text{ 2}}.{\text{2 billion dollars per producer}}^{{0.{\text{5}}}}  \\  \end{aligned}   $$

Having calibrated our model of global production, we compute the sustainable population size if *y* = 20,000 dollars. Let N* denote the size of the sustainable global population. To err on the conservative side of GFN's most recent estimate of 1.6, we assume the global ecological footprint is currently 1.5. That means if the biosphere and the stock of produced capital were stopped on their tracks, their sustainable value would be *K*/1.5, which we denote by K*. Using Eq. (7),

8 $$ (K^{*} )^{{0.{\text{5}}}}  \approx {\text{1}}.{\text{8 billion dollars per producer}}^{{0.{\text{5}}}}  $$

Using Eqs. (6–8), we have

9 $$  (K^{*} )^{{0.5}} \left( {\varphi N^{*} } \right)^{{0.5}}  = \left( {1.8 \times 10^{9} } \right){\text{ }}\left( {\varphi N^{*} } \right)^{{0.5}}  = \left( {20 \times 10^{3} } \right)N^{*}  $$

But φ = 1/2.6. From Eq. (9) it follows that

10 $$ N^{*}  \approx {\text{ 3}}.{\text{3 billion}} $$

Global population was about 3 billion in 1960 (Fig. 3); so, in 3.3 billion we have arrived at a figure that prevailed only about 60 years ago.

The estimate is revealing. Subject to all the caveats we have stressed, it says that if humanity were to find ways to husband the biosphere in a sustainable manner and to bring about economic equality, the human population Earth could support at a living standard of 20,000 dollars is approximately 3.3 billion. If inequality in the distribution of incomes was judged to be inevitable, the figure would be even smaller. It is a simple matter to conduct the exercise with alternative figures for the living standard. We resist doing that.^18^

### Sustainable Living Standard for a Global Population of 9 billion

Equations (8–9) provide us with the tools needed to respond to question (3). Sustainability requires that

11 $$ \left( {1.8 \times 10^{9} } \right){\text{ }}\left( {\varphi N} \right)^{{0.5}}  = Ny $$

But φ = 1/2.6 and *N* = 9 billion. That means Eq. (11) reduces to

12 $$ \left[ {\left( {1.8 \times 10^{9} } \right){\text{ }}\left( {9 \times 10^{9} /2.6} \right)^{{0.5}} } \right]/9 \times 10^{9}  = {\text{ }}y $$

Let y* denote the solution of Eq. (12). Then we have y* ≈ 11,840 dollars at 2011 prices. The figure falls within the range of middle-income countries. But 11,800 dollars at 2011 prices was the global living standard in about year 2000 (Fig. 3). At that time, however, world population was only a little over 6 billion. That 3 billion fewer people did not enjoy a higher living standard should not surprise, because the global stocks of produced capital and human capital were a lot less 20 years ago than it was in 2019 and our model was calibrated with the stocks in year 2019.

## Concluding Remarks

The Anthropocene can be read as being the era when humanity’s ecological footprint vastly exceeds Earth’s ability to meet our demands on a sustainable basis. Because it is met by a diminution of the biosphere, the gap between demand and sustainable supply (the Impact Inequality) is increasing. In this paper, we have followed Barrett et al. (2020) in decomposing humanity’s ecological footprint into global population, per capita global GDP, and the efficiency with which the biosphere’s goods and services are converted into global GDP. Estimates from recent years of the Impact Inequality, world output and its growth rates, and the rate at which natural capital has declined were deployed to study three questions: (1) At what rate must efficiency in resource use rise if the UN’s Sustainable Development Goals for year 2030 are to be sustainable. (2) What would a sustainable figure for world population be if per capita global GDP is to be maintained at an acceptably high level? (3) What living standard could we aspire to if global population was to attain the UN’s near lower-end projection for 2100 of 9 billion?

As the pandemic caused by COVID-19 is still with us, we have based our analysis from the vantage of 2019. As regard (1), the estimates we have obtained under two alternative economic futures tell us that the efficiency parameter α needs to grow at a far higher rate than it has in recent decades in order that the SDG’s, assuming they are reached in 2030, are sustainable.

In our explorations of questions (2–3), we have assumed as a base case that α remains the same as it is today. (Below we show how the analysis can be conducted by assuming alternative future values of α.) In exploring question (2) we have chosen 20,000 dollars in 2011 prices as the basis of the exercise. The figure for *N* we reached is about 3.3 billion, which is about 42% of the present population size. That was the global population in the early 1960s, so it is not an outlandish number. In any event, we have presented the estimate only to show how far off humanity is from where we should probably now be in terms of population size and a sustainable living standard.

We also estimated that that the highest sustainable living standard for a global population of 9 billion would, other things equal, be a bit over 11,800 dollars at 2011 prices. It is a simple matter to re-do the calculations involving questions (2–3) by imagining that the deflator we have used on the current capital structure (the fraction 1/1.5 that was used for moving from Eqs. (7, 8)) is smaller; that is, that α will be higher in the future. We refrain from doing that here.

That human activity since the end of the Second World War has grown faster than ever before is now well appreciated. But formal economic models have not studied its impact on the biosphere. Our aim has been to explore a mode of analysis for doing that, nothing more.^19^

## Appendix

### Two Notions of Inequality

The decomposition of Impact into *N*, *y*, and α has been expressed in aggregate terms. Quite obviously, it is decomposable into income groups. Let *i*, *j*, variously denote households. Households differ according to their incomes, *y*_*i*_, *y*_*j*_, and so on, but they differ as well as regards the efficiency with which they convert the biosphere’s goods and services into income. It is conventional to view inequality in terms of the distribution of household incomes, but here we are interested also in inequality among households in terms of the impact they have on the biosphere. The latter is reflected in the distribution of incomes when corrected for the efficiency with which the biosphere’s goods and services are converted into income (i.e., *y*_*i*_/α_*i*_). And income and income in efficiency units are not the same. We may read *y*_*i*_/α_*i*_ as household *i*’s ecological footprint.

We so label households that *y*_*i*_ < *y*_*i*+1_ for all *i*. There is evidence that households enjoying higher income demand more from the biosphere. That means *y*_*i*_/α_*i*_ < *y*_*i*+1_/α_*i*+1_, for all *i*. A question arises whether the curve *y*_*i*_/α_*i*_ is a convex or concave function of *y*_*i*_. Consider an income interval where the function is convex. An egalitarian redistribution of incomes among households in that interval would lead to a smaller global ecological footprint, implying there is no conflict between income equality and the biosphere’s integrity. But in a concave interval, the reverse holds: egalitarian redistributions of incomes would lead to larger global ecological footprints and society would face a cruel choice between income equality and the biosphere’s integrity (Dasgupta and Dasgupta 2017).^20^ Figure 7, which displays a regression between the ecological footprint of nations and GDP per capita, shows that our demand for the biosphere’s goods and services increases with affluence and development but that the efficiency with which we transform them so increases with affluence that ecological footprint is a concave function of income at all levels of incomes. In short, ecological footprint rises less than proportionately with income. That suggests, ominously, that egalitarian redistributions of incomes lead to larger global ecological footprints, other things the same.

## Footnotes

1 This paper applies the idea of sustainable development in the way it was formulated in what, to the best of our knowledge, was the last scientific publication of which our friend, collaborator, and mentor, Karl-Göran Mäler was a signatory (Barrett et al. 2020). We have tried to frame the questions posed here in a manner that, we hope, would have won his approval. We are grateful to Thomas Viegas of the Bank of England for the estimates underlying Fig. 7 in the Appendix, and to the referees for their most helpful comments and suggestions.

2 We use the terms Nature, the biosphere, the natural environment, and natural capital interchangeably.

3 For simplicity, we imagine that the biosphere’s goods and services can be aggregated (using accounting prices) into a scalar quantity.

4 If it is not possible to aggregate using accounting prices, the ecological footprint would be a vector of demands humanity makes on the biosphere.

5 Only a formal economic model can display quantitatively the mutual influence of technology and institutions on *N*, *y*, and α in a dynamic setting. On this, see Dasgupta (2021, Ch. 4, 13). Investment in family planning and reproductive health is today sorely needed to meet the unmet need for modern contraceptive means among more than 220 million women of reproductive age in the world’s poorest countries. Women’s education is the factor that is advocated in the development literature, but investment in family planning is a neglected, low hanging fruit. Effective policies to reduce future values of *N* from their current projections are of especial importance to Africa, where the fertility rate remains above 4. The UN’s projection of Africa’s population in 2100 is a bit under three times the current population of round 1 billion (UNPD 2019). Such a large increase in numbers in a continent as poor as Africa (GDP per capita in sub-Saharan Africa—just under 4,000 dollars in 2019—is a quarter of per capita global GDP) can be expected to inflict huge stresses on their economies through ecological losses (see Christiaensen 2017; Juma 2019). For a more complete discussion of the place of family planning and reproductive health in sustainable development, see Dasgupta and Dasgupta (2017)

6 We could introduce critical values of the biosphere easily into this framework, as in for example, *G*(*S*) = *rS* [1 − *S*/*K*][(*S* − *L*)/*K*], where *L*, *r*, *K* > 0. Here *L* is the critical value. For *S* > *L*, *G*(*S*) satisfies the conditions assumed in the text.

7 Dasgupta (2021: Ch. 4) collates a rich body of evidence from ecology and the Earth sciences that *S* has declined over the past 70 years.

8 Bifurcations leading to regime shifts can be incorporated into this analysis, as in fn. 5.

9 Barbier and Burgess (2020) offer an excellent discussion of this.

10 In other words, in the late 1960s the global ecological footprint was sustainable, but it is not today. GFN’s estimates are based on data furnished by the United Nations Statistical Office (see Wackernagel and Beyers 2019). It should be noted that as an approximation the authors take *G* to be linear in *S*. GFN has recently reported that the ratio of demand to supply fell to 1.6 in 2020 due to the impacts of the COVID-19 pandemic.

11 While Managi and Kumar (2018) base their work on the UN data base, the questions they ask differ from those asked by the GFN (Wackernagel and Beyers 2019). Moreover, because the Managi-Kumar study includes fossil fuels and minerals, we must assume for our purposes of illustration that the percentage rate of global decline in the accounting value of sub-soil resources equalled the corresponding figure for ecological resources. Using data from different systems of measurement in the numerical calculation we conduct here is a price we must pay for continual neglect of the economics of the biosphere in international organisations. GDP estimates have been refined continually over the decades by countless experts, whereas the human footprint on the biosphere remains of interest only to a handful of people.

12 The commonplace intuition was brought to our attention by a referee.

13 The notion of complementarity we are adopting here differs from the one familiar in the economics of natural resources (Dasgupta and Heal 1979), where it refers to the shape of the isoquants between produced capital and extracted services. Produced capital and natural capital would be found to be complementary to one another in our sense even if the isoquants between produced capital and the flow of Nature’s services in production of output displayed perfect substitutability. And it is complementarity in our sense that matters for an understanding of growth and development. Dasgupta (2021: Ch. 4) contains a complete capital model that brings out the complementarity in our present, stronger sense by tracking production and waste disposal from source to sink.

14 Dasgupta and Dasgupta (2017) posed the same question as we do here, but assumed that humans are mere consumers, that they are not producers of final goods and services.

15 We are not conducting an optimisation exercise, for that would require additional features in the model, such as a social objective function. Dasgupta (2019) studies the problem at length.

16 Unless, of course, the additional output is accompanied by a sufficiently large increase in α.

17 This would be an incorrect assumption in non-stationary states because it ignores differences among sectors in the value that labour adds to production of output.

18 Daily et al. (1994) studied the same problem as ours, but by considering a rate of energy consumption that would offer the average person options to pursue a wide variety of life’s projects and choices. In the early 1990s world population was 5.5 billion and global energy consumption was an annual 13 terawatts (13 trillion watts). The authors assumed that fossil fuels would remain the sources of energy and took it that an annual consumption of 13 terawatts of energy is unsustainable. As we now know from the on-going work of climate scientists, their presumption was right. The authors noted the vast differences in energy use between the world's rich and poor, but on assuming an equitable distribution of energy-use they estimated that a population of 2 billion (world population in the early 1930s) could enjoy a very comfortable life based on an annual 3 terawatts of energy consumption; and that a population of 1.5 billion (world population at the start of the twentieth century) could enjoy an even more comfortable life based on an annual 4.5 terawatts of energy consumption.

19 Cohen (1995) reviewed studies that had estimated Earth’s human carrying capacity. The range he reported was very wide (estimates, in billions, differed by nearly two orders of magnitude); but he did not have at his disposal the ominous biogeochemical signatures that have been uncovered in recent years, nor the findings in MEA (2005a, b, c, d). Dasgupta (2021, Ch. 4* and 13*) presents a complete capital model that can be used to study questions (1)-(3) of this paper in a dynamic setting.
